# Editorial

**Published:** 1860-12

**Authors:** 


### Editorial.

### DEPOSIT OF SECONDARY DENTINE.

The subject of secondary dentine elicits a good deal of attention in the profession. Dr. Rogers, if correctly reported, presented a rather new aspect of the subject at the late meeting of the American Dental Convention. As reported in the *Register*, he says: "Another practice has been to cleanse out a large cavity, then take a piece of gold plate, bending the corners, so as to form feet. Place it in the cavity to form a stool, leaving all empty beneath, and filling with gold on this stool; in removing the filling, found the feet of the stool imbedded in new dentine."

Now, while it has been generally admitted that the pulp, using the term in the wide sense, is capable of depositing dentine, it has not been generally supposed that the circulating apparatus of the toothbone has energy sufficient to carry forward, throw out and organize osseous tissue, beyond the limit of its own extension. But Dr. Rogers is a close observer; and if he has seen new dentine formed under the circumstances above described, he has witnessed a very interesting physiological phenomenon, and has revealed a fact that must revolutionize the views of many of us on this subject; for when *facts* travel, theory must clear the track. But if the circulation through dentine has such power, it is strange that Dr. R. can not trust that of the peroisteum, in favorable cases, to make a little deposit under the stool. It seems, to us, better able to "go to stool" than dentine. W.

---

*The Physicians Pocket Mamorandum: for* 1861. *By* C. H. Cleaveland, M. D. *Cincinnati, B. Frankland, Printer.*

This is truly a *multum in parvo* pocket companion. It sets out with a list of medicines, very minutely classified—classes, divisions, orders, etc. This part of the work is, indeed, unprofitably full and minute. Moreover, it would be improved by more careful or more scientific proof-reading. We open, at random, and find, at the head of page 20, "Ammonia Carbonas," "Potassa Nitrici," and kindred specimens of medical nomenclature ; and on page 21, "Calci Chloridi," and "Zinci Chloridum." And the same thing that is called "Ammonia Carbonas" on page 20, is "Ammoniæ Carbonatis," on page 21. Or, if it is thought these are isolated cases, turn back to page 19, and the first thing we see is "Aurum Chloridi," which means *gold of chloride*, but is there translated "*Chloride of Gold.*" And in the same order we have both "Iodide of Patassium," and "Potassæ Iodidi," and the dose of one is "grs. ij—grs. x," and of the other, "grs. v—grs. x." Such faults as these are as abundant in this department as flies in a summer kitchen.

Another department consists of "Abbreviations used in Prescriptions," and is very full. Another chapter relates to "Accidents and Emergencies." For convenience these are arrayed in alphabetical order. On "Poisons and Antidotes," it is very minute. If this whole department gave artifical respiration a little more prominence, we would like it better. The book contains, also, instructions for Post-mortem Examinations, Preservation and Embalming, and brief suggestions for the prescription of medicines. The main body of the book is composed of pages conveniently arranged for physicians' memorandums. It is well gotten up as to paper, binding, etc., and the practicing physician will find it a desirable pocket companion. Price, $1. W.

### OUR OPINIONS.

We are frequently called upon to give our opinions, upon points of practice, and various professional matters ; this we consider proper enough, provided it is done in a legitimate way and with a proper spirit. If we possess any knowledge on matters of practice, that will be of value to any one, we will gladly impart it—for what we have in that way—is free as the wind, but we would prefer to do it in our own way ; at least, we would rather not write the same opinion over more than *twenty-five times.*

We endeavor, as faithful journalists, to give fully the merits and demerits of any instrument, appliance, process or material, that may be presented to the profession, or that may be of interest. Our opinions in reference to these things are fully and freely set fort in the pages of the Register. And now there are two classes of persons for whom we have a word in regard to this matter; and the first is, those who take the journals, but never read them. We have had some such, write us long letters of inquiry in regard to some subject that we had, perhaps, spent hours in explaining, in, as we supposed, a clear, concise, and understandable manner, upon the pages of the Register; and yet they had not seen it, for in it were the answers to their questions. Now, to such we have to say, if you have read the Register carefully all along, and have farther questions to ask, we shall be glad to have them, and answer them, too, if we can; and if we can not, we will endeavor to get some one who can. The other class is, those who do not take the Register, nor any journal that they will have to pay for, but calculate to keep themselves posted by writing long *penetrating* letters to those whom they suppose can answer their questions; for example, we received a letter, a few days ago, from a dentist, which contained four closely written pages of foolscap, making a great many inquiries about the most common-place subjects and things which have been discussed freely in the Register, and, indeed, in all the journals. Now, we should much prefer, so far as time and labor is concerned, to send him the Register for one year; but we did not do that, nor did we write out a reply to his questions, but gently hinted to him how he could obtain the desired information. T.

---

RICHARDSON'S MECHANICAL DENTISTRY.

This work is just issued by Lindsay & Blakiston, of Philadelphia. It contains four hundred and twenty-seven pages. The mechanical execution of the work is of superior style. We have, as yet, only had time to glance at the work, but are much pleased with its arrangement and the manner in which the subjects are treated. It is more systematically arranged, and treats this branch of Dental practice more fully than has hitherto been done. We propose in the next number of the Register, to make some notes based upon a more minute examination of the work. We hope the work will meet with a ready sale, and large circulation which it richly merits. T.

"MENTAL MISGIVINGS, AND MANY FEARS."

In referring to an article of ours in a preceding number of the Register, our friend, Dr. Pease, says in this number, "And though it is apparent that he has a kind feeling for the nerves, * * * and is willing to lend them a helping hand, and, by trusting them, give them a chance for their lives, it is obvious he does so with some mental misgiving, and many fears." This is true. And we never gave helping hand to a compound fracture, giving the fractured limb a chance for its life, by trusting it, without *some* mental misgivings, and *many* fears. Still, compound fractures are sometimes healed; and exposed pulps are sometimes saved. Dr. Pease's article is of the kind we like to read. It will bear reading several times. But we fear he labors under a very common mistake in believing that a wounded pulp can not heal, except by first intention, without suppuration. Let us suppose that the exposed pulp is wounded slightly. Now, if we apply creosote, or tannin, till it acts to the depth of the wound, we have just the same state of affairs that we would have, if we applied it with the same force to an unwounded pulp. That is, we have a dead, non-putrescible layer on the surface, and living pulp tissue beneath it. The dead, superficial layer, is either a carbolate, or a tannate, the active principle of creosote being *carbolic* acid, and tannin being merely a commercial name for *tannic* acid. But neither the carbolate nor the tannate, thus formed, is putrescible; hence, those who are frightened at the thought of decomposing, dead matter, between the pulp and the filling, may banish their fears. It should be remembered, too, that the dead layer, thus formed must exactly fit the living surface beneath it; consequently, as its texture is everywhere the same, it causes no inequality of pressure. But some anticipate a difficulty here, from an inability of the living parts to adapt themselves to the new state of affairs. They claim that the part acted on by the escharotic, as soon as detatched from the living tissue, is dead matter, and begins to decompose; but they forget that it is dead, as soon as acted on by the escharotic—before it is detached—and that, by its combination with the escharotic agent, it becomes non-putrescible. When detached, it lies there and acts as a non-conductor, under the filling. Others, however, claim that in the process of detachment, there must be effusion, or suppuration. Now, it is evident that soft tissues may and often do, heal either without any effusion, or the effused matters are re-absorbed without producing any inconvenience. When engaged in general practice, our favorite treatment for indolent ulcers of small diameter, was to apply over their entire surface, an active escharotic, capable of forming insoluble precipitates with their albuminous matter, and then to seal them up, perfectly air-tight, and leave them to work out their own salvation. They usually filled up, by a sort of "modeling" process, and without any evidence of either effusion or suppuration between the living parts and the escharotic layer. We have treated ulcers of the pulp (not alveolar abscesses) in the same way, and, in many cases, with like results.

But no one should infer that we regard the exposure of the pulp as a trifling matter. We, too, almost hold our breath, as we approach the pulp cavity, with a cutting instrument. When the pulp is exposed, we have nothing like an assurance that it can be saved alive; and when it is extirpated, there is little assurance that the tooth will retain a healthy vital connection with the general system. In either case, when the constitution is good, and the patient is temperate and *cleanly*, a favorable result may be expected. But every observing dentist knows that a pulpless tooth, even when filled "to the extremity of the fang," is very liable to decline in vitalty, like a tree that has lost its tap-root, till the vital powers of the system regard it as a foreign substance, and undertake its expulsion. When the public mind become so fully awake to the importance of this subject, that all will have their teeth so closely watched, and so promptly attended to, that dentists will have no occasion either to treat or extirpate exposed pulps, then our profession will enjoy its golden age, and will answer the great end of its existence. W.

### THE MALLET.

B., of the American Journal of Dental Science is surprised to see the mallet recommended in the operation of filling teeth. We do not see why he should be surprised, except that he knows nothing about its use for this purpose. He remarks that "nothing can be more unnecessary, and unsafe, than its use in many of the delicate operations of the mouth." Nothing could more clearly exhibit B's ignorance of this *necessary* and very *safe* instrument in the operation of filling teeth, than the above remark.

He will be still more surprised if he will introduce it in practice, at the results obtained.

We have elsewhere spoken of the advantages to be derived from the use of the mallet in filling teeth. We have for almost a year past used this instrument in more than nine-tenths of the cases of filling, and feel so well satisfied with it, that we expect to use it as long as we fill teeth.

Quite a number, within the last year, have introduced it, and we know of none of them who have abandoned it. All who have become familiar with it are much pleased. We advise B. to try it, and he will find the following things : that he can operate with far less fatigue to himself, he will make better fillings, and it will be less objectionable to the patient than hand pressure and that the patient will feel far greater security from accident.

Does B. ever have an instrument slip and wound the soft parts, and give his patient a paroxysm of fidgets; nothing of that kind can occur in the use of the mallet. B.'s sneer at the "tooth carpenters" is wholly gratuitous. Is the surgeon a man carpenter because he uses a saw, a chisel, and a mallet, and boring machines ? Of course he is according to B.

We care but little about the name, or kind of instruments we use, provided they enable us to perform the most perfect operation.

We intend to use, and endeavor to have others use those things that will give the most perfect results, though it should surprise B. T.

